# Relationship between Prolactin, Chronic Kidney Disease, and Cardiovascular Risk

**DOI:** 10.1155/2020/9524839

**Published:** 2020-06-22

**Authors:** Marclébio Dourado, Frederico Cavalcanti, Lucio Vilar, Amaury Cantilino

**Affiliations:** ^1^Nephrology Department, Medical Sciences Center (CCM), Federal University of Pernambuco, Recife, Brazil; ^2^Postgraduate Program in Neuropsychiatry and Behavioral Sciences, Federal University of Pernambuco, Recife, Pernambuco, Brazil; ^3^Nephrology Department, Real Hospital Portugues, Recife, Pernambuco, Brazil

## Abstract

CKD has a high prevalence worldwide, mainly due to its main etiologies—diabetes and hypertension. It has high cardiovascular morbidity and mortality, with traditional risk factors such as atherosclerosis, hypertension, diabetes, smoking, and left ventricular hypertrophy being common. Nontraditional cardiovascular risk factors, such as anemia, hyperparathyroidism, chronic inflammation, and microalbuminuria, are also well studied. Prolactin is a hormone not only related to lactation but also being considered a uremic toxin by some authors. It accumulates with loss of renal function, and it is associated with cardiovascular outcomes in both normal renal function population and CKD population. The purpose of this narrative review is to raise the main common aspects of CKD, prolactinemia, and cardiovascular risk.

## 1. Introduction

Chronic kidney disease (CKD) has high and increasing prevalence in the general population, mainly because the main causes of kidney failure are diabetes mellitus (DM) and hypertension, very common diseases. CKD has high morbidity and is associated with increased cardiovascular mortality, with 5 to 10 million annual deaths worldwide [1].

The so-called traditional cardiovascular risk factors such as DM, hypertension, smoking, left ventricular hypertrophy (LVH), and peripheral vascular disease are very prevalent in this population [2]. However, nontraditional risk factors such as hyperphosphatemia, hyperparathyroidism, inflammation, and anemia are also prevalent in this population. Among these, hyperprolactinemia has received increasing attention in recent years [3].

CKD patients have elevated prolactinemia when compared to the general population, and those with high hormone have higher cardiovascular mortality compared to those with normal prolactinemia [4]. Furthermore, a growing number of biological processes continue to be attributed to prolactin. This is partly due to the presence of its receptor in different tissues at different sites, presenting many cellular responses. These biological processes include insulin resistance, metabolic syndrome, inflammation modulation, endothelial dysfunction, and accelerated atherosclerosis [5].

The aim of this review was to raise the main points regarding prolactin, cardiovascular risks, and CKD.

## 2. Chronic Kidney Disease (CKD)

Chronic kidney disease (CKD) is characterized by progressive and irreversible loss of renal function. It is defined by at least one of the following for more than 3 months: glomerular filtration rate less than 60 mL/min/1.73 m^2^, with or without renal damage markers (albuminuria ≥ 30 mg/24 hours or urinary albuminuria/creatinine ratio ≥ 30 mg/g; persistent glomerular hematuria; persistent electrolyte changes due to tubulopathy; structural abnormalities detected by imaging; previous kidney transplant) [6].

CKD has increasing prevalence worldwide, estimated at 10% of population, being more common in women when dysfunction is mild, and more common in men when dysfunction is severe [7]. Its main etiological factors are diabetes mellitus (DM), systemic arterial hypertension, glomerulopathy, and autosomal dominant polycystic kidney disease (ADPKD), varying depending on the region evaluated. For example, DM is the most prevalent cause of CKD in the United States and Europe, while glomerulopathy are the leading cause in China and Japan [8].

The CKD classification was established in 2012 by the National Kidney Foundation and published in the Kidney Disease Improving Global Outcomes—KDIGO 2013 guidelines. It is widely used for assessment, risk stratification, and follow-up of CKD patients [6]. For example, the more severe the renal dysfunction, the higher the mortality. Five-year survival for a dialysis patient in the United States is approximately 35% and only 25% if the patient is diabetic [1].

CKD is a progressive and silent disease, presenting signs and symptoms only in advanced stages. Therefore, its early diagnosis is very important because, in addition to avoiding a rapid progression to final stages, it is possible to reduce its morbidity and mortality [9].

Besides high morbidity, CKD also has high mortality. Even those patients in renal replacement therapy (RRT) also have high death rates, much higher than certain types of cancer [10]. Due to fluid accumulation, cardiac remodeling, uremic cardiomyopathy, and advanced atherosclerosis, the main cause of mortality in patients with renal failure is cardiovascular [11].

## 3. CKD and Cardiovascular Disease

At all stages, regardless of type of treatment or CKD etiology, cardiovascular disease is the leading cause of death in these patients [12]. Both reduced glomerular filtration (GFR) and increased proteinuria, markers of CKD, increase the risk of cardiovascular disease.

In a large meta-analysis study in which 1,234,182 CKD patients were compared with controls with normal estimated GFR, having estimated GFR reduction to 60, 45, and 15 mL/min/1.73 m^2^ increased mortality due to all causes in 18%, 57%, and 214%, respectively [13].

In the central pathogenesis of heart disease in CKD patients are arterial and myocardial remodeling [14]. As a consequence of volume overload, pressure overload, retention of uremic toxins, vascular calcification, and endothelial dysfunction, these patients present with accelerated atherosclerosis and left ventricular hypertrophy [15].

Therefore, traditional cardiovascular risk factors such as hypertension, LVH, DM, smoking, dyslipidemia, and metabolic syndrome are very prevalent in this population and are widely studied [9]. However, it is important to remind that in CKD patients there are nontraditional cardiovascular risk factors.

For example, vascular calcification is observed even in young adults on dialysis, who do not have associated hypertension, smoking, or dyslipidemia [16]. It is also important to mention that vascular calcification in CKD patients may occur differently from the classical intima layer calcium deposition due to atherosclerosis. In these patients, there may also be deposition in the middle layer as a result of a phenotypic shift from smooth muscle cells to osteoblast-like cells, a phenomenon induced by hyperphosphatemia, hypercalcemia, and hyperparathyroidism [17].

Another nontraditional cardiovascular risk factor common in CKD patients is anemia. Low hemoglobin levels and relative erythropoietin deficiency lead to increased cardiac output, reduced myocardial oxygen supply, oxidative stress, and cardiomyocyte apoptosis. Therefore, anemia is a risk factor for the development and progression of LVH, heart failure, and mortality [18].

Elevated albuminuria (above 30 mg in 24-hour urine) is also a risk factor associated with cardiovascular disease, regardless of presence or absence of diabetes. Although the mechanism by which albuminuria is associated with cardiovascular disease is not well understood, it appears to be a sign that vasculature, particularly the endothelium, is damaged. For example, in diabetic patients, the degree of coronary endothelial dysfunction appears to be higher in those with moderately increased albuminuria [19].

In addition to the nontraditional factors mentioned above, prolactin (PRL) also stands out. This hormone accumulates in the blood with loss of renal function and is associated with cardiovascular outcomes, being considered a uremic toxin [20].

## 4. Prolactin

Prolactin (PRL) is a polypeptide hormone synthesized and secreted by lactotrophic cells of the anterior pituitary gland. The main role assigned to PRL is to stimulate proliferation and differentiation of breast cells needed for lactation by acting on its transmembrane receptor [5].

The size of PRL is heterogeneous in terms of circulating molecular forms. The predominant form in healthy individuals and patients with hyperprolactinemia is monomeric, with a molecular weight of 23 kDa. It can also be dimeric (*big* prolactin, 45–60 kDa) or macro (*big-big* prolactin, 150–170 kDa), both corresponding to less than 20% of the total circulating PRL [21].

Hyperprolactinemia is the most common hypothalamic-pituitary-adrenal axis endocrine alteration. It has several etiologies (Table 1), which can be subdivided into three categories: physiological, pharmacological, and systemic diseases. These include any pathology of the selar region and endocrine and nonendocrine systemic diseases. The most common cause among systemic diseases is prolactinoma, which is present in 50% of patients [22].

PRL was initially described as a lactation-stimulating hormone in mammals. However, in recent years, other actions called “nonpituitary prolactin expressions” have been reported, with over 300 actions at different sites in different species.

A growing number of biological processes continue to be attributed to prolactin. This is partly due to the presence of its receptor in different tissues with multiple intracellular domains, providing a structural basis that can signal to multiple kinases at different sites. Therefore, activation of these signaling pathways may produce different cellular responses.

These biological processes include insulin resistance, metabolic syndrome, inflammation modulation, endothelial dysfunction, and accelerated atherosclerosis [5].

Hyperglycemia has been demonstrated in men and women with hyperprolactinemia due to the direct effects of prolactin on Langerhans islet growth and insulin production [23]. Elevated PRL also has an effect on blood glucose, increasing peripheral insulin resistance [24]. Prolactinoma patients are at higher risk for hyperglycemia, accompanied by obesity and insulin resistance [25]. Due to these effects, bromocriptine, a dopaminergic agonist that inhibits prolactin secretion, was approved in the United States in 2011 for the treatment of type-2 diabetes mellitus [26].

Another metabolic effect that elevated PRL causes is lipid profile, increasing LDL cholesterol and triglycerides, while reducing HDL [27]. A clinical study showed improvement in the metabolic profile of 38 patients with prolactinomas treated with cabergoline, a dopamine receptor agonist that normalizes serum prolactin levels [28]. Another study showed as secondary outcome improved metabolic profile (reduction in LDL, triglycerides, fasting glucose, body mass index (BMI), and waist circumference) in patients with prolactinomas treated with cabergoline for six months [29].

Prolactin is also described as one of the numerous mediators of communication between the neuroendocrine system and the immune system [30]. The PRL receptor is a member of the type 1 cytokine superfamily and is widely expressed in the immune system, including lymphocytes, monocytes, macrophages, natural killer cells, and thymic epithelial cells [31]. Therefore, PRL acts as a hormone and also as a cytokine, participating in several immunomodulatory activities. At endothelial level, PRL stimulates the adhesion of mononuclear cells to endothelial cells in response to inflammatory cytokines [32]. In a recent study, it was found that hyperprolactinemia is associated with activity in autoimmune diseases such as rheumatoid arthritis, systemic lupus erythematosus, scleroderma, and multiple sclerosis [33].

## 5. Prolactin and Cardiovascular Events

In addition to effects described above, serum PRL levels demonstrate an association with several cardiovascular effects. In a study of early menopausal women, PRL, even at normal levels, was found to correlate with the Heart Score of the European Society of Cardiology, a composite index that predicts mortality within 10 years, raising the hypothesis that prolactin may play a role in accelerated arteriosclerosis, affecting blood pressure and stiffness [34].

A study that included 35 hyperprolactinemic patients with untreated pituitary adenomas and 36 healthy controls found that mean carotid intima layer thickness, capillary blood glucose, insulin resistance (HOMA-IR), and ultrasensitive C-reactive protein were significantly higher in patients with elevated PRL, demonstrating that hyperprolactinemia is associated with preclinical atherosclerosis and metabolic abnormalities [35]. Another similar case-control study, this time with 31 prolactinoma patients and 60 controls, demonstrated increased carotid intima-media thickness, with increased insulin resistance, inflammation, and endothelial dysfunction in those with elevated prolactin [36].

In a study with rats, it was demonstrated that different plasma PRL levels have opposite effects: slightly high PRL causes decrease in blood pressure (BP) caused by increased nitric oxide (NO) production, whereas higher hormonal elevations lead to increased BP associated with heart failure due to decreased NO production [37, 38].

Another interesting cardiovascular effect described in association with hyperprolactinemia is peripartum cardiomyopathy (PPCM). PPCM is a rare disease associated with late pregnancy or the postpartum period, marked by severe systolic dysfunction leading to reduced ejection fraction and symptoms of heart failure [2]. It has been shown that in these patients, for reasons yet unknown, PRL cleavage occurs from its 23 kDa form to a 16 kDa form by cathepsin-D. This 16 kDa PRL induces endothelial cell apoptosis, vasoconstriction, reduced metabolism, and cardiomyocyte function, leading to PPCM [39]. In 2010, a pilot study using standard bromocriptine-associated treatment in women with PPCM demonstrated that the group receiving bromocriptine had an improvement in the ejection fraction at 6 months compared to the standard therapy group [40].

An interesting study in patients undergoing endarterectomy found that both fibrotic layer and atherosclerotic plaque macrophages had PRL receptors. In the same study, it was also shown that PRL receptor expression was associated with the atherosclerotic lesion stage—more unstable plaques showed higher receptor expression. Thus, the study raised the hypothesis that PRL has a modulatory effect on atherogenesis [41].

A cohort study evaluating 3,929 individuals (1,946 men and 1,983 women) aged 20–81 years for 10.1 years (with a total of 38,231 person-years) showed, after multivariate analysis, positive association of serum PRL levels with cardiovascular mortality, being the first study to report this positive association [3].

Another more recent cohort study evaluating prolactinemia and incidence of cardiovascular risk found no association between them, but it should be noted that patients with elevated prolactin (above 30 mg/dL) were excluded ((average prolactin levels were normal in women (11.9 mg/dL) and men (8.0 mg/dL) [42]. In this study, each 5 mg/dL PRL increase in men, even at normal levels, was associated with significant increase in hypertension and DM.

## 6. Prolactin and CKD

Kidneys play an important role in endocrine regulation, not only producing hormones such as erythropoietin and renin, but also acting on the metabolism of others such as insulin, cortisol, and prolactin. Therefore, CKD patients have numerous endocrine dysfunctions, with changes in feedback loops, reduced transport of protein-bound hormones, and reduced metabolism and hormone elimination [43].

Regarding to PRL in patients with renal dysfunction, its accumulation occurs by several mechanisms. One of the main mechanisms is reduction in its metabolism [44]. Another important one is increased PRL secretion by lactotrophs in the uremic state—reduced availability of dopamine in the brain directly stimulates PRL secretion [45].

Thus, hyperprolactinemia in these patients becomes very prevalent, ranging from 30% in CKD early stages to 65% in those on hemodialysis [4]. In a study of 31 hemodialysis patients, 30 peritoneal dialysis patients, 30 renal transplant recipients, and 72 controls, prolactin was significantly higher in those on dialysis, with statistically significant difference compared to those transplanted and controls who had prolactin at normal levels. Importantly, hyperprolactinemia observed in CKD patients is not associated with the presence of macroprolactin isoforms [46].

Even with a greatly increased prevalence of CKD, the clinical diagnosis of hyperprolactinemia in this population is difficult. Signs and symptoms of elevated PRL are confused with some manifestations of CKD itself such as oligomenorrhea, amenorrhea, decreased libido, erectile dysfunction, infertility, and osteoporosis. Galactorrhea and gynecomastia are suggestive signs but have limited sensitivity for diagnosis in this population [43].

Dialysis therapies, such as conventional hemodialysis and peritoneal dialysis, do not normalize prolactinemia levels [4]. This is because dialytic therapies generally do not promote effective removal of medium-sized molecules, such as prolactin. Even in frequent hemodialysis, 6 or more times a week, prolactinemia reduction was not observed, as demonstrated in a study with 177 patients on daily dialysis and 60 on nocturnal hemodialysis [47]. High-flow capillary hemodiafiltration (HDF) improves the clearance of molecules up to 25 kDa, sometimes up to 50 kDa, resulting in a reduction in prolactinemia. However, it was evidenced that a few hours after HDF session, PRL levels returned to prehemodialysis values [48].

An interesting cohort evaluating 457 nondialysis CKD patients and 173 hemodialysis patients assessed endothelial function, arterial stiffness, cardiovascular outcomes, and prolactinemia. In nondialysis patients, it was observed a 27% increased risk of cardiovascular events for each 10 ng/mL PRL elevation, as well as a correlation with arterial stiffness. In those on hemodialysis, cardiovascular and all-cause mortality increased by 12% and 15%, respectively, with each 10 ng/mL PRL increase, as well as correlation of prolactinemia with pulse wave velocity [4].

Renal transplantation and consequent improvement in glomerular filtration result in normalization of serum PRL levels [49]. In a study of 14 men and 7 women evaluating PRL at 3 moments (immediately before transplantation and 8 days and 6 months after), sustained normalization of serum hormone levels was observed [50]. Studies using dopaminergic agonists (cabergoline and bromocriptine) are scarce in CKD population, but in those with clinical indication (such as galactorrhea or hypogonadism), their use seems to be safe [51, 52].

## 7. Conclusions

Chronic kidney disease is quite prevalent worldwide, with high cardiovascular mortality. Despite the high prevalence of hyperprolactinemia in this population, there is uncertainty about implications of this condition in patients with CKD, especially with regard to cardiovascular effects. Some authors suggested that prolactin could be a uremic toxin but until now it cannot be concluded whether hyperprolactinemia is a cardiovascular risk factor or only an intermediate in a major pathophysiological pathway. Further studies are needed because if causality between hyperprolactinemia and cardiovascular mortality is demonstrated in CKD patients, this could be a potential therapeutic target.

## Figures and Tables

**Table 1:** Main causes of hyperprolactinemia.

Causes of hyperprolactinemia	Examples
Physiological	Pregnancy
	Breastfeeding
	Stress
	Physical activity
	Breast stimulation

Pharmacological (main drugs)	Risperidone, quetiapine, olanzapine, haloperidol, loxapine, clomipramine, amitriptyline, fluoxetine, citalopram, paroxetine, phenytoin, metoclopramide, domperidone, ranitidine, methyldopa, verapamil, labetalol, morphine, cocaine, sibutramine

Diseases of the selar region	Lactotrophic adenomas (prolactinomas)
	Hypothalamic tumors
	Sarcoidosis, histiocytosis

Endocrine systemic diseases	Hypothyroidism
	Adrenal insufficiency

Nonendocrine systemic diseases	Chronic kidney disease
	Hepatical cirrhosis
